# Chemotherapy-induced small extracellular vesicles prime the pre-metastatic niche to accelerate neuroblastoma metastasis

**DOI:** 10.1016/j.gendis.2023.05.016

**Published:** 2023-07-04

**Authors:** Carson A. Wills, Xiaoming Liu, Longgui Chen, Yuanjun Zhao, Zhenqiu Liu, Vladimir S. Spiegelman, Jeffrey Sundstrom, Hong-Gang Wang

**Affiliations:** a Department of Pediatrics, Division of Pediatric Hematology and Oncology, The Pennsylvania State University College of Medicine, PA 17033, USA; b Department of Ophthalmology, The Pennsylvania State University College of Medicine, PA 17033, USA; c Department of Public Health Sciences, The Pennsylvania State University College of Medicine, PA 17033, USA

Neuroblastoma is the most common extracranial solid tumor in children, accounting for more than 10% of cancer-related deaths in this population. The standard of care for patients diagnosed with medium-to high-risk neuroblastoma, including those who present with metastatic disease, is preoperative induction chemotherapy.^1^ However, approximately 40% of patients experience relapse or recurrence despite treatment, and evidence suggests that certain chemotherapeutic drugs may promote metastasis through poorly-understood mechanisms.^2^ Recent research has highlighted the role of small extracellular vesicles (sEVs) in regulating cancer progression and metastasis through the transfer of cellular material.^3^ In this study, we investigated how DNA-damaging chemotherapeutic drugs affected the secretion and contents of neuroblastoma sEVs and how these sEVs impacted metastasis. We demonstrate that chemotherapy increases the secretion of neuroblastoma sEVs and alters their protein content, leading to accelerated hepatic metastasis in both immune-competent and -deficient mouse models. Additionally, we identify sEV-associated pentraxin 3 (PTX3), an acute-phase inflammatory glycoprotein, and the tissue-type plasminogen activator (PLAT), a serine protease, as critical factors involved in the formation of the pre-metastatic niche with a STAT3-associated inflammatory gene signature.

To determine the effect of chemotherapy treatment on neuroblastoma sEV secretion, we selected concentrations of DXR that inhibit less than 10% of cell viability *in vitro* in the human SK-N-AS and the mouse 9464D neuroblastoma cell lines (Fig. S1A). We isolated sEVs from the conditioned cell culture media of cells treated for 24 h with DXR or DMSO, the vehicle control, using differential ultracentrifugation. We confirmed that isolated vesicles displayed the characteristic size, morphology, and surface markers of sEVs (tetraspanins CD63 and CD9) using electron microscopy (Fig. 1A), immunoblotting (Fig. 1B), and nanoparticle tracking analysis (Fig. S1B). We found that low-dose DXR treatment significantly up-regulated both the number of sEVs and the amount of sEV protein secreted per cell *in vitro* (Fig. 1C, D) and that the amount of protein secreted per sEV was not significantly altered by DXR (Fig. S1C). We also confirmed that this phenotype is not unique to DXR by isolating sEVs from SK-N-AS cells treated with etoposide and daunorubicin (Fig. S1D–G).

**Figure 1:**
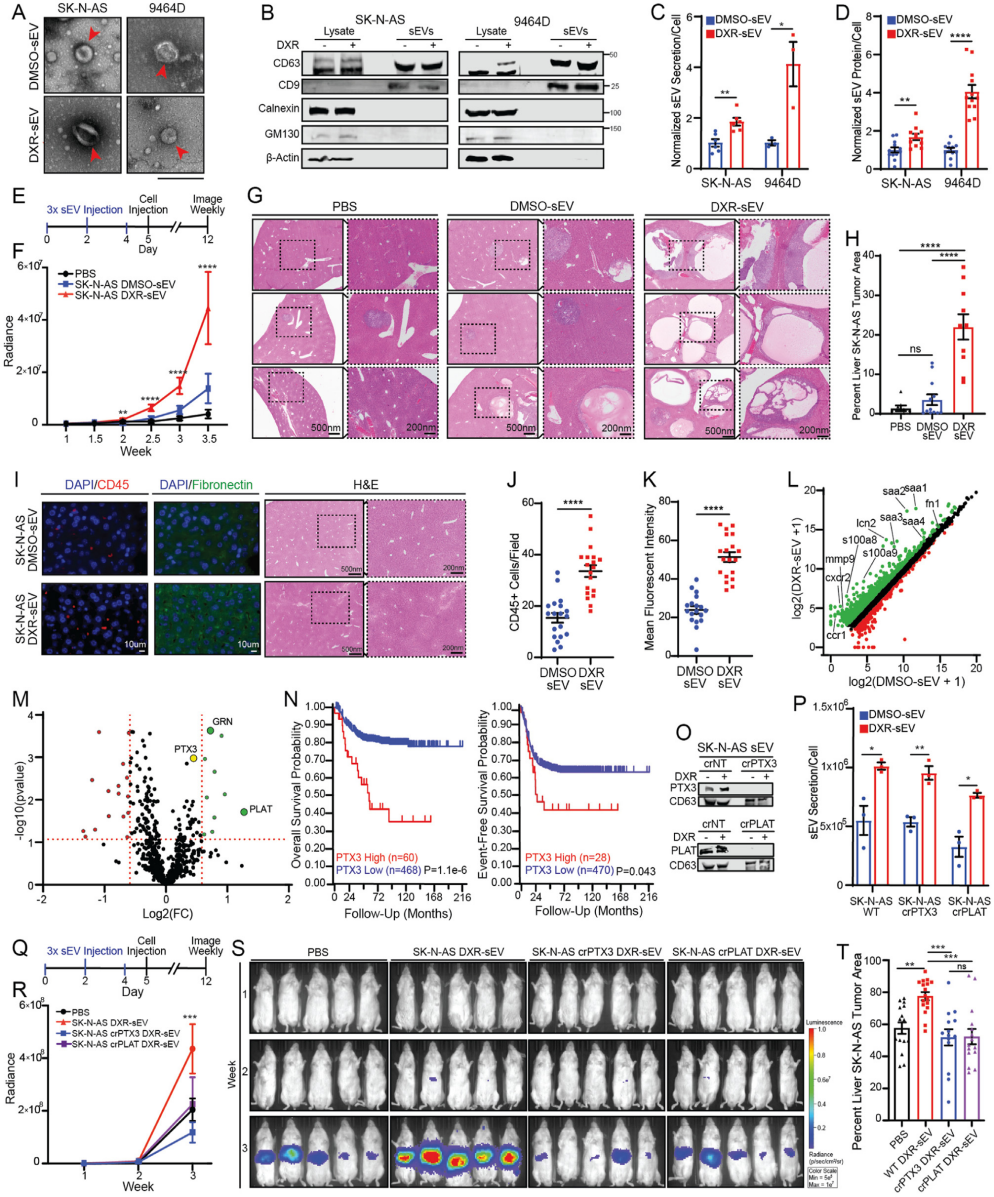
Chemotherapy induces neuroblastoma sEV-associated PTX3 and PLAT secretion and thus promotes hepatic pre-metastatic niche formation and accelerates metastasis. **(A)** Representative electron microscopy images of SK-N-AS and 9464D DMSO-sEVs and DXR-sEVs. Scale bar = 200 nm. **(B)** Immunoblot of SK-N-AS and 9464D sEVs with characteristic positive and negative EV markers. **(C)** Quantification of sEV secretion per cell normalized to DMSO-sEV average for each cell line. SK-N-AS, *n* = 3; 9464D, *n* = 3; Student's *t* test; ^∗^*P* < 0.05, ^∗∗^*P* < 0.01. **(D)** Quantification of sEV protein secretion per cell normalized to DMSO-sEV average for each cell line. SK-N-AS DMSO, *n* = 12; SK-N-AS DXR, *n* = 11; 9464D, *n* = 12; Student's *t* test; ^∗∗^*P* < 0.01, ^∗∗∗∗^*P* < 0.001. **(E)** Experimental design for priming the pre-metastatic niche mouse experiments. (F) Region of interest quantification of SK-N-AS whole-body metastatic tumor radiance. The data represented two independent experiments. PBS, *n* = 7; SK-N-AS DMSO-sEV, *n* = 10; SK-N-AS DXR-sEV, *n* = 10; Two-way ANOVA; mean ± SEM; ^∗∗^*P* < 0.01, ^∗∗∗∗^*P* < 0.001. **(G)** Representative histology images of SK-N-AS liver metastatic lesions. Scale bar: 500 nm for left figures and 200 nm for insets. **(H)** Average percentage of liver area covered by SK-N-AS tumor, quantified using ImageJ. The data represented two independent experiments. PBS, *n* = 7; SK-N-AS DMSO-sEV, *n* = 10; SK-N-AS DXR-sEV, *n* = 10; one-way ANOVA; mean ± SEM; ^∗∗∗∗^*P* < 0.001; ns, not significant. **(I)** Representative images of liver tissues derived from mice treated three times with SK-N-AS DMSO-sEVs or DXR-sEVs stained with indicated pre-metastatic niche markers. H&E images of corresponding liver tissue. Scale bar: 500 nm for left figures and 200 nm for insets. **(J)** Quantification of visible CD45^+^ cells per field. Two liver sections were imaged and 10 random fields per slide were obtained. Student's *t* test; mean ± SEM; ^∗∗∗∗^*P* < 0.001. **(K)** Quantification of mean fibronectin fluorescent intensity using ImageJ. Two liver sections were imaged and 10 random fields per slide were obtained. Student's *t* test; mean ± SEM; ^∗∗∗∗^*P* < 0.001. **(L)** Differentially expressed genes in liver tissue following treatment with SK-N-AS DMSO-sEVs or DXR-sEVs. Green indicates the up-regulation in livers of mice treated with DXR-sEVs compared to DMSO-sEVs, and red indicates the down-regulation in livers of mice treated with DXR-sEVs compared to DMSO-sEVs. **(M)** Volcano plot of differentially expressed sEV proteins. Green indicates the up-regulation in SK-N-AS DXR-sEVs compared to DMSO-sEVs, and red indicates the down-regulation in DXR-sEVs compared to DMSO-sEVs. **(N)** GSE49710 dataset overall and event-free survival probability for neuroblastoma patients stratified by PTX3 expression. **(O)** Immunoblot of SK-N-AS DMSO-sEVs and DXR-sEVs derived from crNT, crPTX3, and crPLAT cells probed for indicated proteins. **(P)** Quantification of sEV secretion per cell. *n* = 3 for all conditions. Student's *t* test; mean ± SEM; ^∗^*P* < 0.05, ^∗∗^*P* < 0.01. **(Q)** Pre-metastatic niche mouse model experimental design. **(R)** Region of interest quantification of SK-N-AS whole-body metastatic tumor radiance. The data represented one experiment. PBS, *n* = 10; SK-N-AS DXR-sEV, *n* = 10; SK-N-AS crPTX3 DXR-sEV, *n* = 9; SK-N-AS crPLAT DXR-sEV, *n* = 10; Two-way ANOVA; mean ± SEM; ^∗∗∗^*P* < 0.005. **(S)** Representative IVIS images of whole-body SK-N-AS metastatic luciferase signal in mice from indicated treatment groups. **(T)** Average percentage of liver area covered by SK-N-AS tumor, quantified using ImageJ. The data represented two independent experiments. PBS, *n* = 16; SK-N-AS WT DXR-sEV, *n* = 17; SK-N-AS crPTX3 DXR-sEV, *n* = 14; SK-N-AS crPLAT DXR-sEV, *n* = 15; one-way ANOVA; mean ± SEM; ^∗∗^*P* < 0.01, ^∗∗∗^*P* < 0.005; ns, not significant.

We previously reported that DXR-elicited sEVs (DXR-sEVs) from breast cancer cells accelerate pulmonary metastasis by priming the pre-metastatic niche.^4^ To determine whether neuroblastoma sEVs affect metastasis, we first used whole-organ fluorescent imaging to establish that both SK-N-AS and 9464D DMSO- and DXR-sEVs primarily localize to the liver, lungs, and bone marrow *in vivo* within the first 24 h following tail vein injection (Fig. S2). To determine whether uptake of DXR-sEVs in these tissues alters the metastatic capacity of neuroblastoma cells, we injected NSG or C57BL/6 mice three times over the course of five days with 10 μg of DXR- or DMSO-sEVs derived from SK-N-AS or 9464D cells respectively or an equivalent volume of PBS. On the fifth day, mice were injected via the tail vein with SK-N-AS or 9464D cells expressing a luciferase reporter construct to experimentally model metastatic dissemination (Fig. 1E). We found that whole-body metastatic tumor growth was significantly enhanced in mice pre-treated with DXR-sEVs compared to mice pre-treated with DMSO-sEVs or PBS in both immunodeficient and immunocompetent neuroblastoma tumor models (Fig. 1F; Fig. S3A–C). Quantification of liver tumor burden in mice inoculated with SK-N-AS cells (Fig. 1G, H; Fig. S3D) or lung and liver tumor burden in mice inoculated with 9464D cells (Fig. S3E) at the experimental endpoint confirmed that metastatic tumor growth was significantly enhanced in mice pre-treated with DXR-sEVs compared to DMSO-sEVs or PBS.

To confirm that DXR-sEV treatment promotes the formation of the pre-metastatic niche, we isolated liver tissue from mice treated three times with DMSO-sEVs or DXR-sEVs derived from SK-N-AS cells and performed immunofluorescent imaging to quantify bone marrow-derived cell accumulation and stromal deposition, two hallmarks of the pre-metastatic niche. Indeed, we observed a significant accumulation of both CD45^+^ cells and fibronectin deposition in liver tissue isolated from mice treated with DXR-sEVs compared to DMSO-sEVs (Fig. 1I–K).

To elucidate the mechanism by which DXR-sEVs prime the pre-metastatic niche, we performed RNA sequencing of whole liver tissue isolated from NSG mice treated three times with SK-N-AS DMSO-sEVs or DXR-sEVs. We found that genes involved in acute phase inflammatory response pathways were significantly up-regulated in the livers of mice treated with DXR-sEVs compared to DMSO-sEVs (Fig. 1L; Fig. S4A–C). Importantly, the expression of inflammatory genes known to be involved in the formation of the liver pre-metastatic niche was significantly up-regulated by DXR-sEV treatment (Fig. S4C). Notably, most of these genes are downstream of the STAT3 signaling pathway, suggesting that hepatic uptake of DXR-sEVs may accelerate metastasis through STAT3-mediated inflammation.

To determine how DXR-sEV cargo up-regulates an acute inflammatory response in the liver, we performed an unbiased proteomic analysis of SK-N-AS DMSO-sEVs and DXR-sEVs (Fig. 1M; Fig. S4D). Of the proteins that were significantly up-regulated in DXR-sEVs compared to DMSO-sEVs, PTX3, PLAT, and granulin (GRN) particularly attracted our attention. We previously identified sEV-associated PTX3 as a critical regulator of chemotherapy-induced breast cancer metastasis.^4^ We also found that high levels of PTX3 correlated with poor overall and event-free survival in neuroblastoma patients (Fig. 1N). Although the levels of PLAT and GRN expression are inversely correlated with neuroblastoma patient survival (Fig. S5A, B), PLAT has a complex role in cancer progression.^5^

To determine whether the uptake of these proteins in the liver primes the pre-metastatic niche, we first confirmed that they were up-regulated in SK-N-AS DXR-sEVs compared to DMSO-sEVs. We then used CRISPR/Cas9 to knock out (KO) each gene in SK-N-AS cells and confirmed that the protein of interest was absent from isolated sEVs (Fig. 1O; Fig. S5G). We confirmed that KO sEVs are morphologically similar to WT sEVs and determined that while DXR treatment significantly up-regulated sEV secretion in both cell lines, neither *PTX3* nor *PLAT* KO altered sEV secretion per cell compared to WT (Fig. 1P; Fig. S5C, D). We also used density gradient ultracentrifugation and dot plot analysis to confirm that PTX3 and PLAT were truly sEV-associated proteins that primarily localize to the sEV surface (Fig. S5E, F).

To determine whether KO suppressed sEV-induced metastasis *in vivo,* we repeated our pre-metastatic niche model by treating mice three times over the course of five days with DXR-sEVs derived from WT or KO cells, or an equivalent volume of PBS, followed by tail vein injection of WT SK-N-AS cells (Fig. 1Q). Notably, we found that mice pre-treated with either crPTX3 or crPLAT DXR-sEVs demonstrated a significant decrease in whole-body metastasis compared to mice pre-treated with WT DXR-sEVs or PBS (Fig. 1R, S), while mice pre-treated with crGRN DXR-sEVs demonstrated no significant difference in metastasis compared to WT DXR-sEVs (Fig. S6A, B). Histologic quantification of liver metastatic tumor burden at the conclusion of the experiment corroborated these findings (Fig. 1T; Fig. S6C, D).

Collectively, these data suggest a model whereby DXR treatment up-regulates the secretion of sEVs in neuroblastoma and differentially regulates the packaging of their protein cargo. The uptake of these pro-metastatic DXR-sEVs in the liver triggers an acute inflammatory response, creating a favorable environment for neuroblastoma cell colonization. PTX3 and PLAT are critically involved in sEV-induced neuroblastoma metastasis, while GRN plays no significant role. These findings hold promise for the development of more effective therapeutic options for high-risk neuroblastoma patients.

## Author contributions

C.A.W. was responsible for study design, experimentation, data analysis, manuscript preparation, and manuscript revision. X.L. carried out experimentation and data analysis with assistance from L.C., Y.Z., and Z.L. V.S. and J.S. assisted with study design, procurement of materials, and data analysis. H.G.W. directed and supervised the project, and was involved in study design, manuscript preparation, and manuscript revision.

## Conflict of interests

The authors declare no conflict of interests.

## Funding

This work was supported in part by Four Diamonds, Lois High Berstler Research Endowment Fund, and 10.13039/100000002National Institutes of Health Grant 5T32CA060395.
