# Supplementary material for: Chemotherapy-induced small extracellular vesicles prime the pre-metastatic niche to accelerate neuroblastoma metastasis
Source: Genes Dis. 2023 Jul 4;11(4):101017. doi: 10.1016/j.gendis.2023.05.016 (PMC10940767; doi:10.1016/j.gendis.2023.05.016)
Supplement: Multimedia component 1 [file mmc1.docx]

**SUPPLEMENTARY DATA**

**Materials and Methods**

*Cell lines*

The human neuroblastoma cell line SK-N-AS (CRL-2137) was purchased from ATCC, and the mouse neuroblastoma cell line 9464D was a gift from Dr. Paul Sondel (University of Wisconsin, Madison, Wisconsin, USA). SK-N-AS luciferase reporter cells were generated with a pCDH-SV40-Luc2 construct and selected with hygromycin (200μg/mL). To make 9464D luciferase reporter cells, 9464D cells were transduced with pCDH-EF1-Luc2-P2A-tdTomato (Addgene #72486) lentivirus and sorted into single clones. Twenty-one validated single clones were pooled to create the stable 9464D Luc-tdTomato cell line. CRISPR/Cas9 knockouts were performed by sub-cloning three single guide RNAs (gRNA) targeting *PTX3* (5’- CCTCTGCCAGTGGCTCCAC-3’, 5’-CTCCAAATTTATCACAACAG-3’, 5’- TGCAGTTATGGGACACAGCA-3’), *PLAT* (5’-CAAACATAATTACTGCCGGT-3’, 5’- CCTCCTTTGATGCGAAACTG-3’, 5’-GAGCCAAGGTGTTTCCAACGG-3’), or *GRN* (5’- ATCGACCATAACACAGCACG-3’, 5’-CCCTGCCCAGAGGACTAACA-3’, 5’- CTGCTGCCGTCTACAGTCGG-3’) into pLenti-CRISPR-V2 (Addgene 52961). Lentiviral packaging was performed in 293T cells and SK-N-AS cells were transduced with pLenti-CRISPR-PTX3, pLenti-CRISPR-PLAT, and pLenti-CRISPR-GRN. Puromycin selection (1μg/mL) was used to isolate pooled knockout cells, which were screened using immunoblotting for gene disruption. All cell lines used in this study were periodically authenticated by mycoplasma testing, morphologic inspection, and STR analysis.

*Chemicals and antibodies*

Doxorubicin (S1208) and Etoposide (S1225) were obtained from Selleck Chemicals, and Daunorubicin (D8809) was obtained from Sigma-Aldrich. Antibodies used for immunoblotting were CD63 (Abcam ab68418), CD9 (Santa Cruz sc-13118), Calnexin (Abcam ab22595), GM130 (BD Biosciences 610822), ß-Actin (Sigma A5441), PTX3 (Santa Cruz sc-373951), and tPA (Fisher Scientific AF7449SP).

*Small extracellular vesicle isolation*

sEV-depleted FBS was made by centrifuging heat-inactivated FBS twice at 120,000 RCF for 12 hours at 4°C and filtering the supernatant through a 0.2μM filter. DXR-sEVs were isolated by collecting conditioned media from SK-N-AS and 9464D cells cultured for 24 hours in DMEM supplemented with 10% (v/v) heat-inactivated sEV-depleted FBS and treated with 350nM DXR (SK-N-AS cells) or 1.8μM DXR (9464D cells) for 24 hours. Control DMSO-sEVs were isolated from the conditioned media of cells treated with a volume of DMSO equivalent to 10% the volume of DXR. The conditioned media was centrifuged at 500 RCF for 10 minutes at 4°C to remove cells and large cell debris. The supernatant was then collected, filtered through a 0.2μM syringe filter (VWR 28145-501), and concentrated using Pierce 100K MWCO protein concentrators (Thermo Fisher 88533) by centrifuging at 4000 RCF and 4°C. The concentrated supernatant was centrifuged at 10,000 RCF for 20 minutes at 4°C to remove larger microvesicles and apoptotic bodies. The supernatant was centrifuged at 120,000 RCF for 2.5 hours at 4°C, washed once with PBS and centrifuged for an additional 2.5 hours at 120,000 RCF, then washed a second time with PBS and centrifuged for 12 hours at 120,000 RCF. The sEV pellet was resuspended in PBS and stored at -20°C. Nanoparticle tracking analysis and transmission electron microscopy were performed to quantify and image sEVs.

*Tail vein injection metastasis mouse model*

All animal studies were performed according to guidelines established by the Institutional Animal Care and Use Committee (IACUC) at the Penn State College of Medicine (Hershey, PA). Male and female 6-8-week-old immunodeficient NOD SCID Gamma (NSG, Jackson 005557) mice were used for all experiments involving the human SK-N-AS cell line, and male and female immunocompetent C57BL/6J (Jackson 000664) mice were used for all experiments involving the mouse 9464D cell line. Priming of the pre-metastatic niche assays were performed by injecting mice via the tail vein with 10μg sEVs in 100μL PBS, or an equivalent volume of PBS, on days 0, 2, and 4, followed by tail vein injection of 2.5 x 10^5^ SK-N-AS or 9464D cells on day 5. Metastatic tumor growth was quantified by imaging mice weekly or biweekly using an IVIS Lumina III *in vivo* bioluminescent imaging system. Mice were anesthetized using 2.5% isoflurane (IsoSol Isoflurane, USP, VEDCO) and injected with 5μL/gram body weight 30mg/mL D-Luciferin (Syd Labs MB000102-R70170) dissolved in PBS 5 minutes prior to imaging. Photon flux was calculated using region of interest (ROI) measurements around the whole body to quantify metastatic tumor growth. At the experimental endpoint, mice were euthanized, and lung and liver tissue were harvested for *ex vivo* analysis and histology.

*Immunohistochemistry*

Liver tissue derived from mice at the experimental endpoint was fixed in formalin, processed in an automated Tissue-Tek VIP processor, paraffin-embedded, and sectioned into 6μm thick slices. Sections were stained with hematoxylin and eosin for histopathologic analysis. Quantification of liver metastatic tumor burden was performed by taking multiple images of each liver slide, quantifying the total tumor area in each liver section, and dividing by the total tissue area of that section using ImageJ software. The tumor area for each section was averaged to determine an overall tumor area for each liver sample.

*Immunofluorescent imaging*

Fixed liver tissue taken at the experimental endpoint was deparaffinized in xylene and ethanol, and pre-heated sodium citrate buffer (10mM, pH 6.0) was added to each slide and incubated at 95^o^C for 15 minutes in a heater-hybridizer. The slides were then drained and incubated for 5 minutes in cooled buffer, before returning to the heater for 5 minutes. This cycle was repeated once more, and then the slides were cooled on the bench for 30 minutes in buffer. The slides were then washed twice for 5 minutes in 1xTBS with 0.1% Tween-20 and then blocked in 5% serum with 1% BSA in 1xTBS for 2 hours at room temperature. The slides were then drained and incubated overnight with the following primary antibody at 4^o^C: Fibronectin (Abcam ab2413), CD45 (Fisher Scientific NC0158520). The following day the slides were rinsed twice for 5 minutes in 1xTBS with 0.1% Tween-20 and the following fluorophore-conjugated secondary antibody diluted in 1xTBS with 1% BSA were applied to the slide and incubated for one hour at room temperature: Alexa Fluor 488 Goat Anti-Rabbit (1:1000, Invitrogen A-11008) and Alexa Fluor 594 Chicken Anti-Rat (1:1000, Invitrogen A-21471). The slides were then rinsed twice for 5 minutes in 1xTBS with 0.1% Tween-20 and ProLong Gold antifade mounting reagent with DAPI (Invitrogen, P36935) was applied to each slide. Slides were imaged using an Olympus IX61 microscope. CD45+ cells were counted in 10 random fields of vision per slide, and fibronectin mean fluorescent intensity was quantified in 10 random fields of vision per slide using ImageJ software.

*RNA Sequencing*

For RNA sequencing analysis, male NSG mice were injected via the tail vein with 50μg DXR- sEVs or DMSO-sEVs in 100μL PBS on days 0, 2, and 4. Mice were sacrificed on day 5 and whole liver tissue was isolated and flash frozen in liquid nitrogen, and RNA was extracted using TRIzol. cDNA libraries were generated with the QuantSeq 3’ mRNA-Seq Library Prep Kit (Lexogen), checked for size distribution using BioAnalyzer (Agilent), diluted to 2nM, and run on an Illumina HiSeq 2500. Adaptors were trimmed from raw FASTQ files using the BBDuk tool. The trimmed FASTQ files were mapped against the mm10 mouse reference genome using the STAR aligner. The aligned reads were quantified with htseq-count (https://htseq.readthedocs.io/en/master/count.html). The differential expression of gene (DEG) analysis was performed using Deseq2 (https://bioconductor.org/packages/release/bioc/html/DESeq2.html) R package. The DEGs (log2FC > 0.5 with an adjusted p-value (FDR) < 0.1) were subjected to the Metascape database (http://metascape.org) for analysis of enriched pathways and processes with Gene Ontology (GO) biological processes, Kyoto Encyclopedia of Genes and Genomes (KEGG) pathways, and Reactome Gene Sets. Heatmaps were generated using Heatmapper (http://heatmapper.ca) to visualize differentially expressed genes.

*Quantitative PCR*

Quantitative PCR (qPCR) analysis was performed on RNA samples isolated from the livers of mice treated three times with DMSO-sEVs or DXR-sEVs, as described above. cDNA synthesis was made from 2.5ug liver RNA using a GoScript Reverse Transcriptase kit (Promega, A5001). qPCR reactions were prepared using iQ SYBR Green Supermix (Bio-Rad, 1708880) and run using a QuantStudio12K Flex system in the Penn State Genome Sciences Core. The sequences for the following primers were obtained from the Harvard PrimerBank: SAA1, 5’- ACAGCTTGGTGACTGACTGA-3’; SAA2, 5’-TGGCTGGAAAGATGGAGACAA-3’; SAA3, 5’-TGCCATCATTCTTTGCATCTTGA-3’; SAA4, 5’-CTCTGTTCTTTGTTCCTGGGAG-3’; CCR1, 5’-CTCATGCAGCATAGGAGGCTT-3’; LCN2; 5’-TGGCCCTGAGTGTCATGTG-3’; CCL6, 5’-GCTGGCCTCATACAAGAAATGG-3’, S100A8; 5’- AAATCACCATGCCCTCTACAAG-3’; S100A9, 5’-ATACTCTAGGAAGGAAGGACACC- 3’; GAPDH, 5’-AGGTCGGTGTGAACGGATTTG-3’.

*Proteomic analysis*

For mass spectrometry analysis, 100μg sEVs were resuspended in PBS and quantified using tandem mass spectrometry at the University of Michigan. Proteins were filtered against a human database using a false discovery rate of less than 5%, and pathway analysis was performed using Advaita iPathway Guide (Advaita Bioinformatics).

*Cell viability assays*

Cell viability was measured using CellTiter-Glo Luminescent Cell Viability Assay (Promega G7570) according to manufacturer’s instructions in 96-well plates and imaged using a CLARIOstar (BMG Labtech). All data were normalized to their nontreated controls.

*Small extracellular vesicle uptake assay*

To quantify sEV uptake, sEVs were labeled with Vybrant DiD Cell Labeling Solution (Thermo Fisher V22887) by resuspending sEV pellets in 1mM Vybrant DiD and incubating at room temperature for 10 minutes. Samples were then centrifuged at 120,000 RCF for 2 hours, washed once in PBS and centrifuged at 120,000 RCF for another 2 hours at 4^o^C. Labeled pellets were then resuspended in PBS and stored at -20^o^C prior to use. For in vivo uptake studies, mice were injected via the tail vein with 10μg labeled sEVs in 100μL, or an equivalent volume of PBS. 24 hours after injection, mice were euthanized, whole organs were isolated, and fluorescent imaging was performed using an IVIS Lumina III (excitation 640nm, emission cy5.5). Radiant efficiency was calculated using region of interest (ROI) measurements around each individual organ and normalized to PBS-treated controls.

*Dot plot*

50μg intact sEVs were dotted onto a 0.2μm nitrocellulose membrane and dried for one hour. The membrane was blocked in Odyssey blocking buffer for one hour at room temperature, then probed with primary antibody at 4^o^C overnight with or without 1% Tween-20 (Fischer Scientific BP337-500), which was used to disrupt the sEV lipid membrane and expose internal proteins. Membranes were washed three times in blocking buffer and probed with fluorescent secondary antibody for one-hour rocking at room temperature with or without 1% Tween-20 and 0.1% 10% sodium dodecyl sulfate (SDS). The membrane was washed three times in blocking buffer and signal was detected using a LI-COR Odyssey CLx Imager.

*Statistical analysis*

GraphPad Prism was used for statistical analysis of data. Two-tailed student t-tests were used for single comparisons, and groups differences were evaluated using one-way or two-way ANOVA. Statistical significance was set to P < 0.05.

**Supplementary Figures**


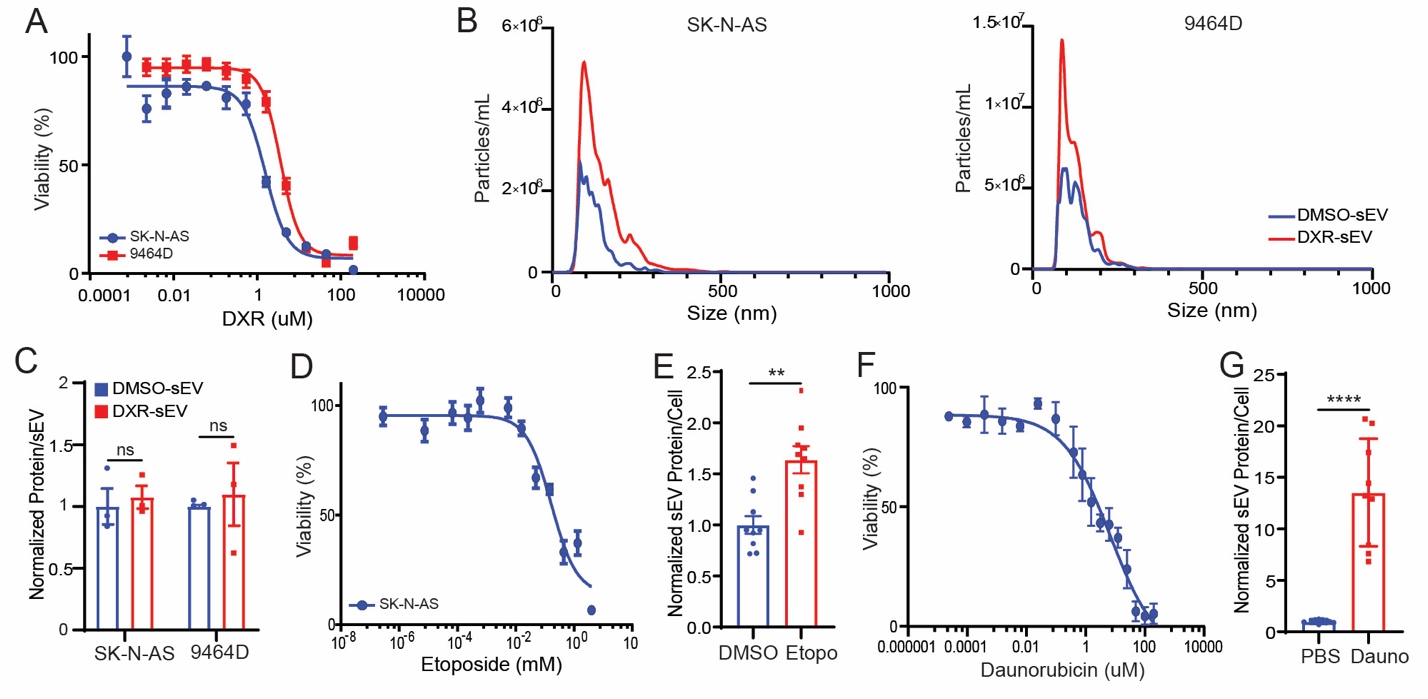


**Figure S1.** DNA-damaging chemotherapeutic drugs upregulate neuroblastoma small extracellular vesicle secretion.

(**A**) Cell titer glo 24h viability assay of DXR-treated SK-N-AS and 9464D cells. SK-N-AS n=6, 9464D n=6. (**B**) Representative NTA graphs of SK-N-AS and 9464D DMSO-sEVs and DXR-sEVs. (**C**) Protein secreted per sEV normalized to DMSO-sEV average per cell line. SK-N-AS n=3, 9464D n=3. Student’s t- test, ns- not significant. (**D**) Cell titer glo viability 24h assay of SK-N-AS cells treated with etoposide, n=3. (**E**) Normalized sEV protein secreted per cell following 24 hours of DMSO or etoposide treatment. Samples normalized to DMSO average for each replicate. Mean ± SD, DMSO n=9, Etoposide n=9, Student’s t-test, **p<0.01. (**F**) Cell titer glo 24h viability assay of SK-N-AS cells treated with daunorubicin, n=10. (**G**) Normalized sEV protein secreted per cell following 24 hours of PBS or daunorubicin treatment. Samples normalized to PBS average for each replicate. Mean ± SD, DMSO n=9, Etoposide n=9, Student’s t-test, ****p<0.001.


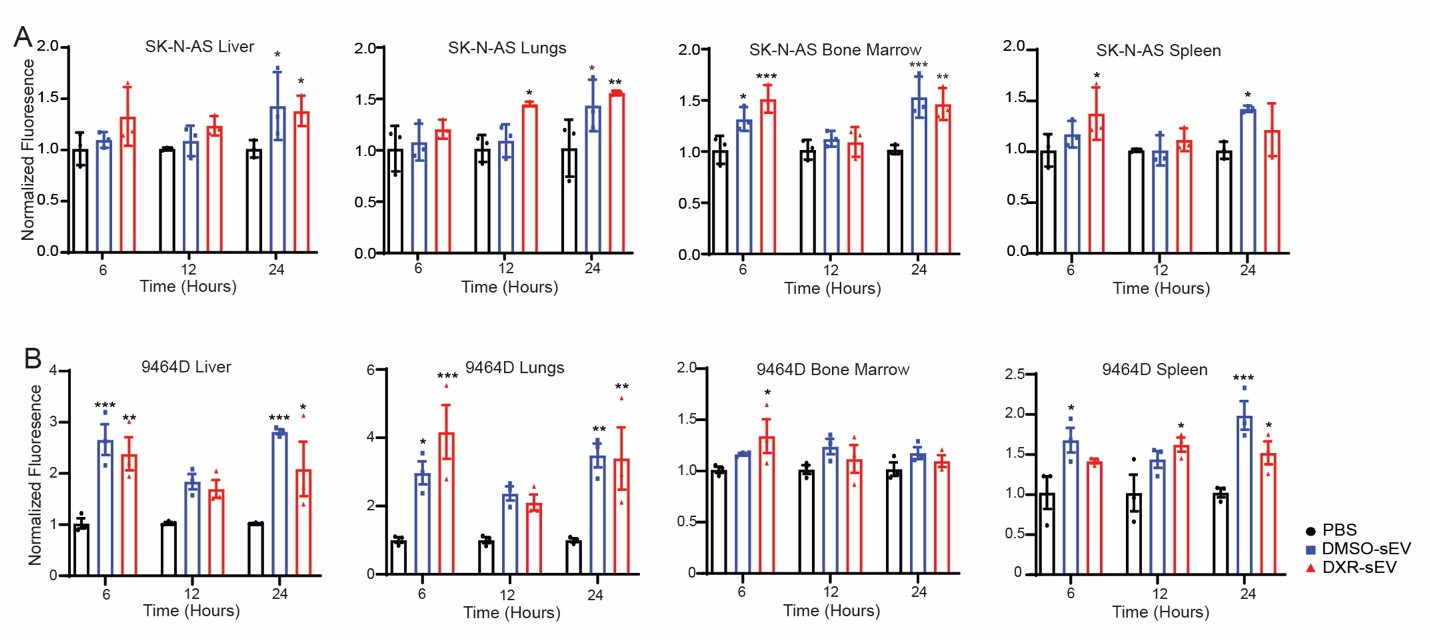


**Figure S2.** Neuroblastoma sEVs localize to the liver, lungs, bone marrow, and spleen *in vivo.*

Quantification of whole-organ fluorescence at indicated timepoints following tail vein injection of PBS or 10ug SK-N-AS DMSO-sEV or DXR-sEVs (**A**) into male NSG mice; or 10ug 9464D DMSO-sEVs or DXR-sEVs (**B**) into male and female C57BL/6 mice. Liver, lung, bone marrow, and spleen, normalized to PBS average for each organ. Two-way ANOVA, mean ± SD, n=3 for all timepoints, *p<0.05, **p<0.01, ***p<0.005, ****p<0.001.


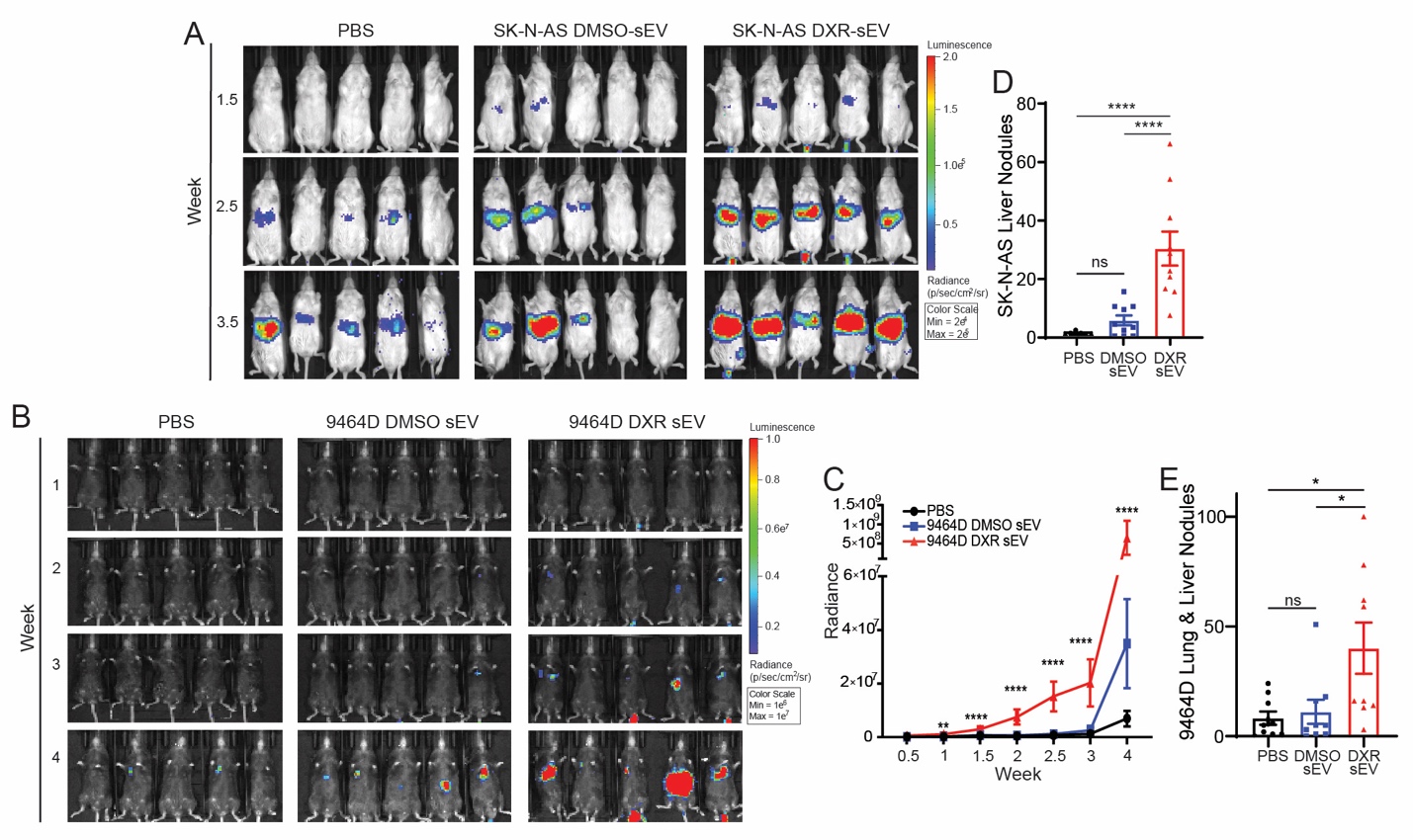


**Figure S3.** Neuroblastoma DXR-sEVs accelerate metastasis in both an immunocompetent and an immunodeficient mouse model.

(**A**) Representative IVIS images of whole-body SK-N-AS metastatic luciferase signal in mice from indicated treatment groups. (**B**) Representative IVIS images of 9464D whole-body metastatic luciferase signal in mice from indicated treatment groups. (**C**) Region of interest quantification of 9464D whole- body metastatic tumor radiance. Data represent one experiment. PBS n=9, 9464D DMSO-sEV n= 9, 9464D DXR-sEV n= 9. Two-way ANOVA, mean ± SEM, **p<0.01. ****p<0.001. (**D**) Quantification of total visible SK-N-AS liver tumor nodules. Data represents two independent experiments. PBS n=7, SK-N-AS DMSO-sEV n= 10, SK-N-AS DXR-sEV n= 10. One-way ANOVA, mean ± SEM, ****p<0.001. (**E**) Quantification of total visible liver and lung 9464D tumor nodules. Data represent one experiment. PBS n=9, 9464D DMSO-sEV n= 9, 9464D DXR-sEV n= 9. One-way ANOVA, mean ± SEM, *p<0.05.


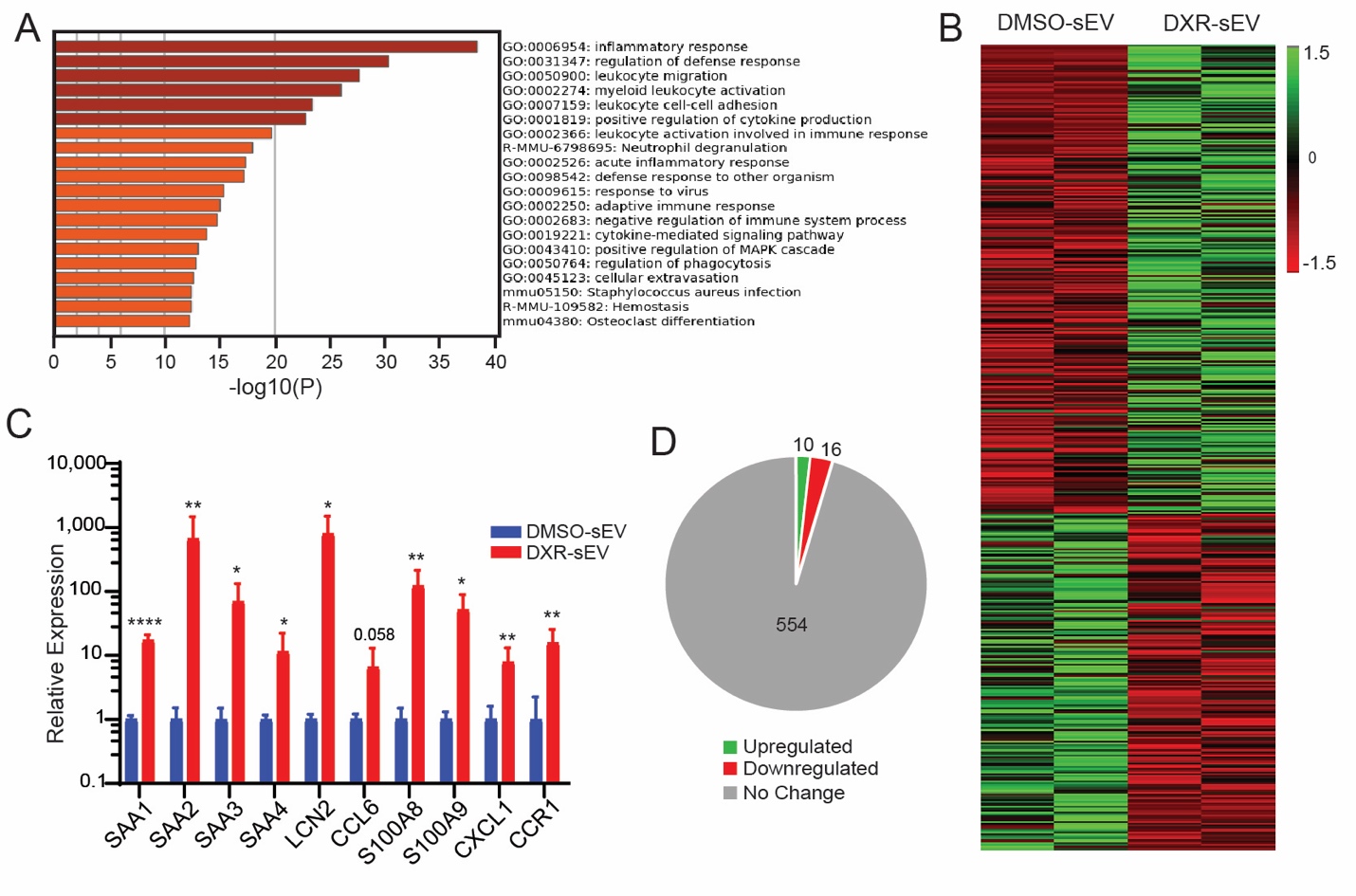


**Figure S4.** SK-N-AS DXR-sEVs upregulate inflammatory pathways in the liver.

(**A**) Metascape GO pathway analysis of top upregulated pathways in liver tissue of mice treated with SK-N-AS DXR-sEVs compared to DMSO-sEVs. (**B**) Heatmap of significantly up and downregulated genes in liver. N=2 for each treatment. (**C**) Relative mRNA expression of STAT3 signaling pathway genes in livers of mice treated with SK-N-AS DXR-sEVs (red) or DMSO-sEVs (blue). (**D**) 580 proteins were identified in SK-N-AS sEVs, 10 were significantly upregulated in DXR-sEVs compared to DMSO-sEVs, 16 were significantly downregulated in DXR-sEVs compared to DMSO-sEVs.

**
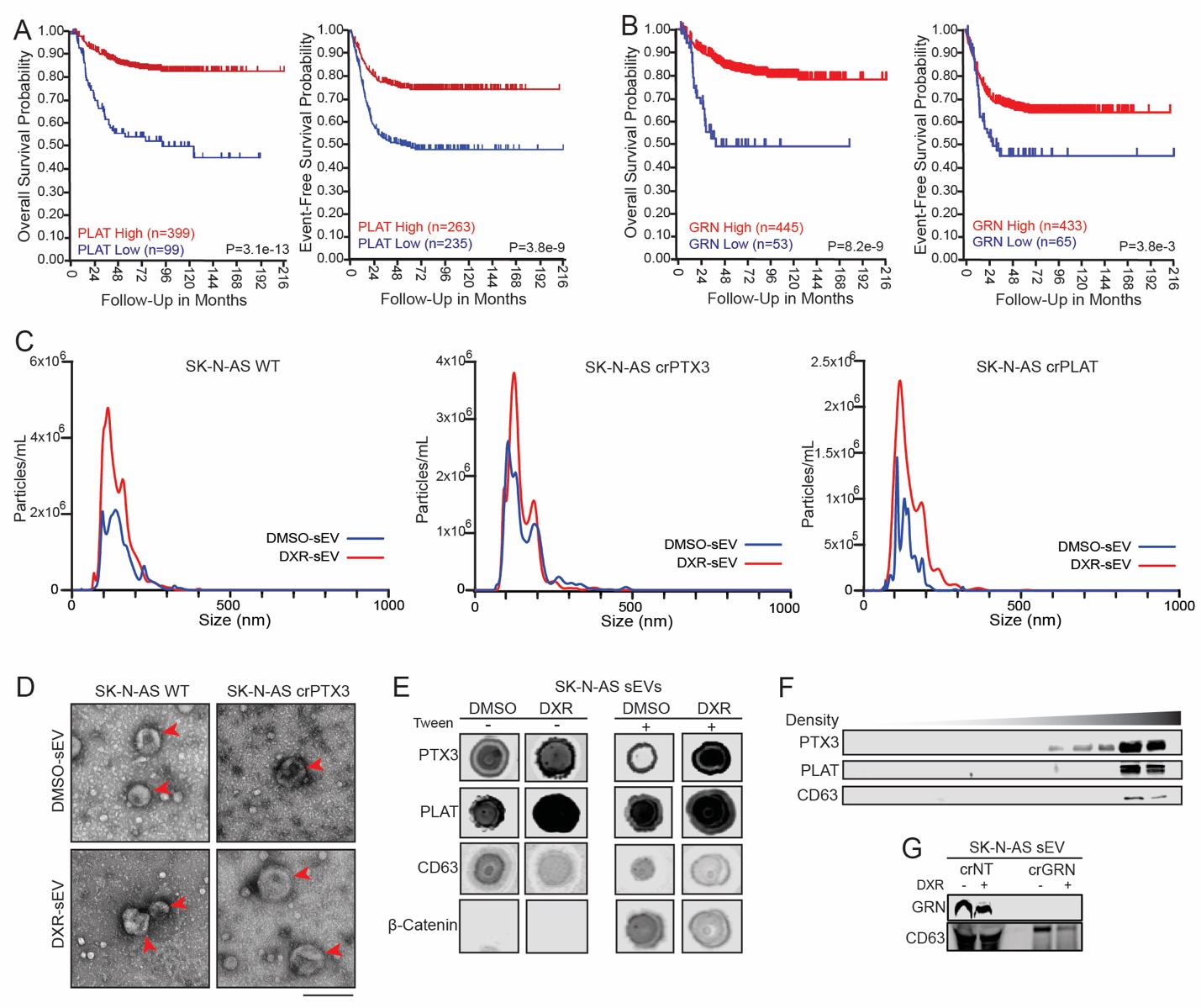
**

**Figure S5.** Knockout of *PTX3* and *PLAT* do not affect neuroblastoma sEV secretion.

(**A**) GSE49710 dataset overall and event-free survival probability for neuroblastoma patients stratified by PLAT gene expression. (**B**) GSE49710 dataset overall and event-free survival probability for neuroblastoma patients stratified by GRN gene expression. (**C**) Representative NTA graphs of SK-N-AS WT, crPTX3, and crPLAT DMSO-sEVs and DXR-sEVs. (**D**) Representative EM images of vesicles derived from indicated cell lines. Scale bar=200nm. (**E**) Dot plot of SK-N-AS DMSO-sEVs and DXR-sEVs probed for indicated proteins in the presence or absence of Tween-20 detergent. (**F**) Density gradient ultracentrifugation of SK-N-AS DXR-sEVs probed for indicated proteins. (**G**) Immunoblot of SK-N-AS DMSO-sEVs and DXR-sEVs derived from crNT and crGRN cells probed for indicated proteins.


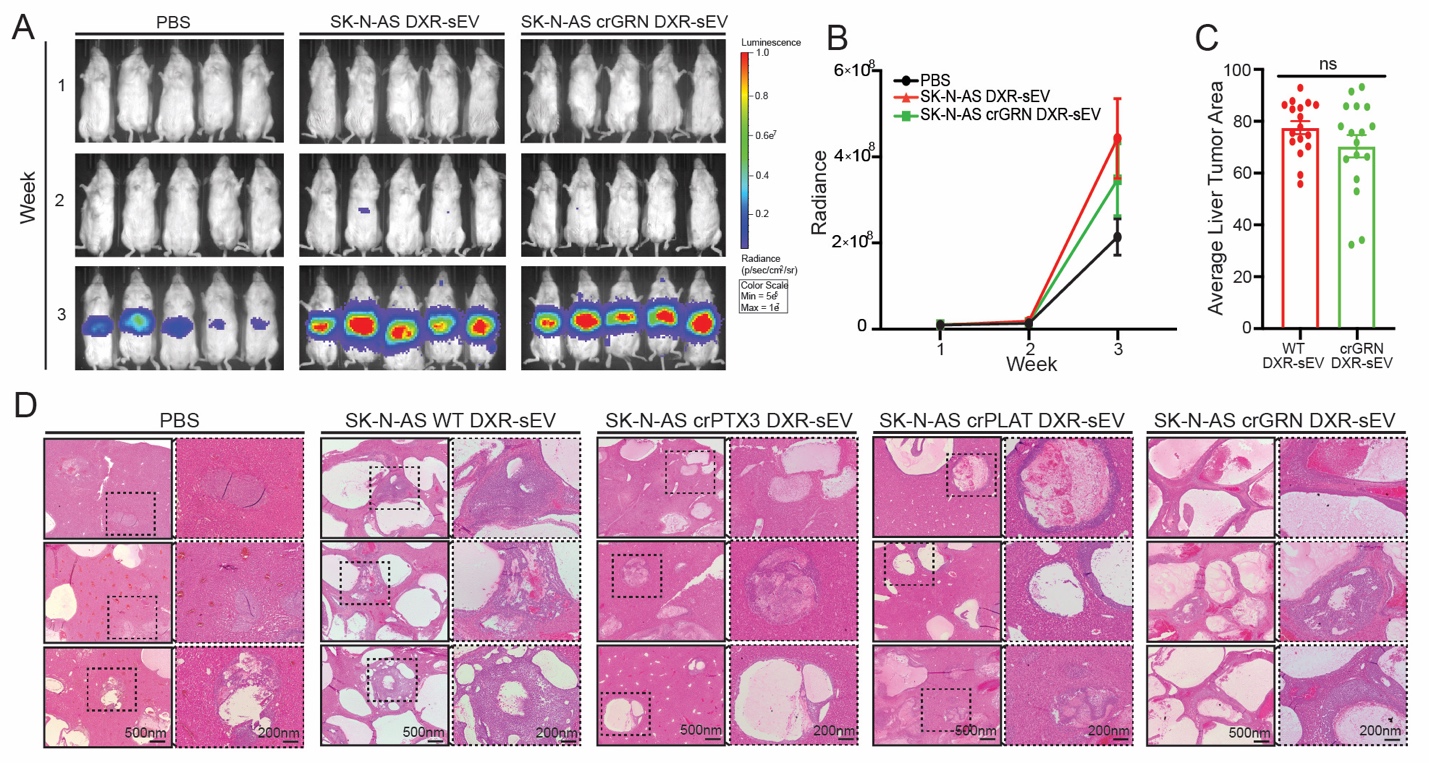


**Figure S6.** SK-N-AS sEV-associated GRN does not affect neuroblastoma metastasis.

(**A**) Representative IVIS images of whole-body SK-N-AS metastatic luciferase signal in mice from indicated treatment groups. (**B**) Region of interest quantification of SK-N-AS whole-body metastatic tumor radiance. Data represent one experiment. PBS n=10, SK-N-AS DXR-sEV n= 10, SK-N- AS crGRN DXR-sEV n=10. Two-way ANOVA, mean ± SEM. (**C**) Quantification of average liver metastatic burden. Data represent one experiment, student’s t-test, mean ± SEM, SK-N-AS WT DXR-sEV n=17, SK-N-AS crGRN DXR-sEV n=17, ns not significant. (**D**) Representative histology images of SK-N-AS liver metastatic lesions.
